# Modeling Adhesive Wear in Asperity and Rough Surface Contacts: A Review

**DOI:** 10.3390/ma15196855

**Published:** 2022-10-02

**Authors:** Haibo Zhang, Roman Goltsberg, Izhak Etsion

**Affiliations:** 1 School of Mechanical Engineering, Beijing Institute of Technology, Beijing 100081, China; 2 Department of Mechanical Engineering, Technion, Haifa 32000, Israel

**Keywords:** adhesive wear, Archard law, asperity contact, finite element method, numerical models, Rabinowicz criterion, wear mechanisms

## Abstract

Wear is one of the most fundamental topics in tribology and adhesive wear is argued as the least avoidable wear type. Numerical techniques have allowed advances in more realistic simulations of adhesive wear mechanisms and promoted our understanding of it. This paper reviews the classic work on wear modeling by Archard and Rabinowicz, followed by a comprehensive summary of the adhesive wear numerical models and techniques based on physical parameters. The studies on wear mechanisms at the asperity level and rough surfaces are separately presented. Different models and their key findings are presented according to the method type. The advantages and deficiencies of these models are stated and future work, such as considering more realistic geometries and material properties for adhesive wear modeling, is suggested.

## 1. Introduction

Wear is one of the most fundamental topics in tribology, affecting all aspects of our lives and current technologies in fields such as engineering [1], medical care [2], and environment protection [3] to name a few. The wear process, causing material loss due to relative sliding between contacting surfaces, is generally thought harmful in the majority of practical situations. It can be, however, beneficial in situations such as polishing a surface and writing with a pencil [4]. Among the various types of wear processes, adhesive wear [5] is argued as the least avoidable one [6]. It occurs due to the strong adhesive bonds between contacting and near contacting asperities of interacting surfaces and results in material removal in the form of wear particles which are transferred to the other surface or detached in loose form [4]. The wear particles, leaving pits, voids, cavities, or valleys on the original surface, can be transferred back again which, in a back and forth transfer mode, results in the surface being extremely rough [7]. Moreover, the loose wear particles may become harder third-body abrasive particles, resulting in intensified erosive and abrasive wear [8].

Although the first scientific experimental investigation of wear started as early as in 1803 by Hatchett [9], researchers still have an incomplete understanding of it, i.e., in what process the wear particles form and how to calculate their volume. Holm [10] is probably one of the earliest researchers who attempted to model the adhesive wear mechanism and the following decades saw the emergence of a myriad of empirical or phenomenological models. For instance, Godet [11] discussed two main approaches in developing wear models (see Section 4.14 there): the ones based on empirical data collection or on known physical principles, while Meng et al. [12] reviewed 182 equations of wear predictive equations in the literature. Among these equations, the Archard wear law [13], with the nature of an empirical law, is the ubiquitous one. As early as in 1977, Suh indicated that “The wear rate of metals may be predicted in the near future, based on first principles and fundamental material properties” [14]; however, to date, such a satisfying prediction model has not been reached. Due to the few alternatives, it is not surprising that the long-standing empirical Archard wear equation [13] is still widely used, as concluded in [15].

Accurately modeling the adhesive wear processes is a substantial challenge due to the formidable complexity emerging from a variety of physical and chemical mechanisms at disparate time and length scales [6,16]. This complexity leads to the difficulties in accurately predicting adhesive wear a priori based only on materials’ property data and contact information [17]. As a result, accurate modeling of the adhesive wear mechanisms still remains one of the unsolved problems in tribology.

Thanks to the development of computer simulations, behind which stands the development of computational science and many areas related to surface science, progresses in accurately modeling the adhesive wear process could be made by considering more realistic conditions. Computer simulations, being in a sense experiments on computer via direct numerical techniques, efficiently replicate real-world experiments to some degree, and hence, allow insights into the physical behaviors that are hardly visible in physical experiments. More accurate computer simulations usually require larger computing resources and longer computing time, which can be resolved, although nowadays only to some extent, with the aid of supercomputers and parallel computing techniques.

Various numerical methods with different features have been developed and used in modeling adhesive wear. The finite element method (FEM) and boundary element method (BEM) are two main numerical methods in continuum mechanics on the macroscale, with FEM usually being considered versatile and BEM as efficient. The atomic simulations with molecular dynamics (MD), which model the physical movements of material (classically for atoms or molecules on the microscale) as “particles”, have become a powerful modeling method. The review for these three methods and others used in tribological modeling can be found in Ref. [18].

It is well-known that for rough surfaces, the real contact occurs at the summits of the highest asperities. Greenwood and Williamson [19] proposed the multi-asperity model in which a rough surface is composed of independent multiple asperities having spherical tips at their summits and a statistical distribution of summit heights. Similarly, the adhesive wear of rough surfaces could be understood by incorporating the wear behavior of a single asperity, which should be studied initially, into the multi-asperity model. The procedure in this single-to-multiple asperity model is also applicable even when the rough surface is modeled by a self-affine fractal surface [20,21], as was undertaken, for example, in Ref. [22].

The present review focuses on studies which utilized numerical techniques for physically modeling adhesive wear. The numerical simulations for wear evolution of macroscopic surface morphology implementing empirical wear law are not included, because they do not come close to understanding the mechanisms of the formation or any other events of wear particles. This paper begins with reviewing the classic Archard wear law and Rabinowicz’ criterion for adhesive wear modeling. In the following sections, various numerical simulations on adhesive wear modeling at the asperity level, including the two-dimensional (2D) and three-dimensional (3D) cases, are discussed according to the method type. Then, the works for adhesive wear modeling of rough surfaces are presented and compared.

## 2. Classic Wear Theories

### 2.1. Archard Wear Law

In 1953, Archard [13] generalized Holm’s concept of “atom removal” [10] and proposed an adhesive wear model by assuming a hemispherical wear particle of the same radius, *a*, as that of the asperity contact area formed by fully plastic deformation. Thus, the wear rate (the ratio of wear particle volume, proportional to *a*^3^, to the effective sliding distance, 2*a*) is proportional to the real contact area (proportional to *a*^2^) at the asperity level. For this plastic contact area, the load supported is also proportional to *a*^2^, and thus wear rate is proportional to the load. This argument is then applied to all other contacting asperities of the rough surface, giving a linear relationship between total wear rate and total normal load *P*. Then, a probability factor *K* is applied on each contact asperity to account for the fact that not all asperity encounters result in wear particles. This finally gives the widely used Archard wear law as follows [23]:

(1) $$V=K\frac{P}{H}s$$

where *V* is the wear volume, the probability factor *K* is the wear coefficient, *H* is the hardness of the worn material, and *s* is the sliding distance. The wear coefficient *K*, interpreted as the probability of wear particle formation by Archard, is an empirical parameter and should be determined by wear tests.

Although the Archard wear law, given by Equation (1), became a ubiquitous wear law in the following decades [18], it lacks fundamental physical understanding, as empirical laws essentially do [24,25]. It is applicable only to cases where the test conditions for finding *K* exactly match the real application [26], and *K* usually varies with several orders of magnitude from case to case. The linear dependence of the wear volume on the normal load was found questionable with experimental evidence as early as in 1970 in Ref. [27]. In spite of the approximation to reality, the Archard wear law is still used due to its simplicity as well as lack of any other, more elaborated wear law [28].

### 2.2. Rabinowicz’ Criterion

In 1958, Rabinowicz [29] proposed a criterion for determining the size of loose wear particles, which in fact describes nothing about how a wear particle forms but about whether a formed particle will detach from the surface due to adhesive load. Rabinowicz started by assuming that a formed wear particle, having a hemispherical shape and residual stresses and strains within, induced by a mating surface, is attached to a surface after its mating surface has moved on. The elastic energy $E_{e}$ stored in the wear particle is assumed as 0.1 (interpreted as square of Poisson’s ratio later in Ref. [30]) of the maximum possible deformation energy in the particle during contact and is given by:

(2) $$E_{e}=\frac{1}{10}\cdot \frac{\sigma_{Y}^{2}}{E}\frac{\pi d^{3}}{12}$$

where $\sigma_{Y}$ is the yield stress, *E* is the elastic modulus, and *d* is the diameter of the wear particle. According to Rabinowicz, the detachment of a wear particle from the surface can happen only when the elastic energy *E_e_* is greater than the adhesive energy:

(3) $$\frac{1}{10}\cdot \frac{\sigma_{Y}^{2}}{E}\frac{\pi d^{3}}{12}\geq W\frac{\pi d^{2}}{4}$$

where *W* is the work of adhesion per unit area. Equation (3) can be rewritten as:

(4) $$d\geq d_{c}=\frac{30EW}{\sigma_{Y}^{2}}$$

which determines the critical diameter *d*_c_, above which wear particles can detach.

The principle used in Rabinowicz’ criterion is very simple, which could be the reason that this idea does not attract enough attention and saw almost no development since then. However, despite its simplicity, Rabinowicz’ criterion seems to capture the key concepts as given in Equation (4), which is revealed by recent work with atomic simulations, as will be discussed in Section 3.

## 3. Modeling Adhesive Wear at Asperity Level

As mentioned in Section 1, modeling the adhesive wear mechanism at asperity level is the first step for studying the contact of rough surfaces. This section introduces the relevant works and arranges them into subsections according to the method type and also model dimensions, i.e., 2D or 3D model. One of the reasons for using a 2D model (mostly in a plane strain condition) is that it significantly reduces the computing time. However, in reality, the asperities (as well as the rough surface) are in three dimensions, and thus a 2D model, as a simplification, is analyzed only to provide qualitative understanding for the real 3D case.

### 3.1. Finite Element Method

#### 3.1.1. Two-Dimensional Models

Pointing out the weaknesses in Archard’s model, Suh [31] proposed a qualitative explanation, known as the “delamination theory of wear”, for the adhesive (also for fretting and fatigue, as was claimed) wear mechanism based on dislocation dynamics. Accordingly, wear occurs by delamination of sheets as a result of subsurface deformation, crack nucleation, and crack propagation. Sin and Suh [32] conducted the initial effort on developing a 2D elastic-plastic FE model to quantitatively predict the growth rate of subsurface cracks under a moving asperity (Hertzian) load. In this model, the subsurface crack, with length of 2*c* and at a depth of *d* beneath the surface, was set a priori and its length growth rate was determined by the crack tip deformation caused by the combined compressive and shear loads. It was found that the linear elastic fracture mechanics (LEFM) approach used in previous studies such as [33] is inappropriate due to the large plastic zone at the crack tip. The crack growth was found to be faster with smaller predefined depth *d*, contradicting the experimental observation that cracks are only observed at a finite distance below the surface. This is due to the unphysical predefinition of *d*, which was explained to depend on the state of stress and the metallurgical factors.

Keer et al. [34] modeled the wear particle formation in asperity of 2D elastic-plastic wedges with different tip angles using FEM. They found that a wear particle may be created in one of the three wedge failure modes: shear failure, fracture failure, and slip tongue failure, which agree well with experimental observations of the “slip-tongue” and the wedge in SEM by Kato et al. [35].

Similar 2D FE models were then developed in Refs. [36,37,38,39,40] in the following decades with either an LEFM or elastic-plastic approach to investigate the subsurface crack growth. However, all these works suffer from the need to define a crack a priori, limiting the initial conditions of the simulation.

**Figure 1:**
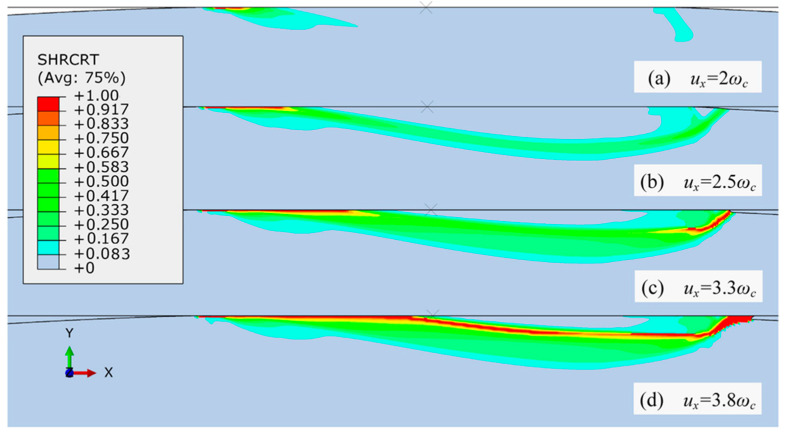
Development of the fracture initiation during tangential loading of *u_x_*. Taken from [41].

Considering that it is difficult to predefine the crack nucleation and propagation due to a nonlinear stress field under varying contact loads, Wu et al. [41] developed a 2D elastic-plastic FE model free of this restriction. In this model, the classic configuration of flattening and then shearing a deformable asperity by a rigid flat was used, with an imposed shear strength of asperity as the upper limit of shear stress at its contact interface. Three different ductile failure criteria for fracture initiation were included and in each criterion the fracture occurs upon the local equivalent plastic strain $\bar{\varepsilon }$ reaching the critical value ${\bar{\varepsilon }}_{f}$. Such criteria enable more realistic fracture prediction due to the link of fracture initiation and stress distribution. For the elastic preloaded contact, Wu et al. [41] found that no noticeable wear particle was generated. For the elastic-plastic preloaded contact, the evolution of fracture is shown in Figure 1, where SHRCRT is the damage parameter and when its value of an element reaches 1 (red color), the fracture criterion is satisfied. They found that the initialization and propagation of a fracture consists of three stages: first, the fracture initiates and propagates at the trailing portion of the contact area; second, an oblique daughter fracture emerges in the leading contact edge; finally, the two fractures propagate and link below the contact area, forming a flake-like wear particle.

Wu et al. [42] further extended the model in Ref. [41] to metallic materials containing inclusions, showing that subsurface micro-cracks, particularly those near inclusions, and their subsequent evolution significantly influence the generation of flake-like wear particles. In Wu et al.’s work [41,42], an element satisfying the fracture criterion was not removed from the mesh but was restricted so that only compressive stresses can be supported, which is unable to model the detachment of a wear particle from the surface.

In the investigation of sliding friction between two cylinders (2D asperities), Mulvihill et al. [43] presented a case showing similar wear particles by using an FE model with material failure criteria, however, without further discussion for the wear behavior.

#### 3.1.2. Three-Dimensional Models

Salib et al. [44] presented an approach for potential adhesive wear particles, on the basis of the model by Brizmer et al. [45] for elastic-plastic spherical contact with a rigid flat under combined normal and tangential loading. The maximum shear strain was used as a criterion to determine the slip interface (subsurface fracture) in the bulk; however, no realistic failure criterion for elements is included and, hence, without occurrence of a crack. This approach predicted the volume of a potential, rather than actual, wear particle.

The authors developed a 3D FE model for adhesive wear of spherical contact [46], eliminating the deficiencies of dimensions and failure criteria above. A full stick contact condition was used to model the strong adhesion at the contact interface. To model the fracture, the realistic failure criteria of ductile material were applied using two steps: the Johnson–Cook (JC) criterion [47] for damage initiation in the first step, and the additional fracture energy criterion [48] for damage evolution in the second step. The elements satisfying the fracture criteria were deleted from the mesh (indicating the slip interface) and the active elements remaining between the flat and slip interface specify the wear particle. The elastic and elastic-plastic preloading were then investigated, both showing flake-like wear particles but with larger thickness for larger normal load. The use of the element deletion technique enables the modeling of the detachment of wear particles; however, unphysical material loss at the slip interface (zero thickness actually) occurs, leading to overestimated interference of the sphere when the fracture evolves.

**Figure 2:**
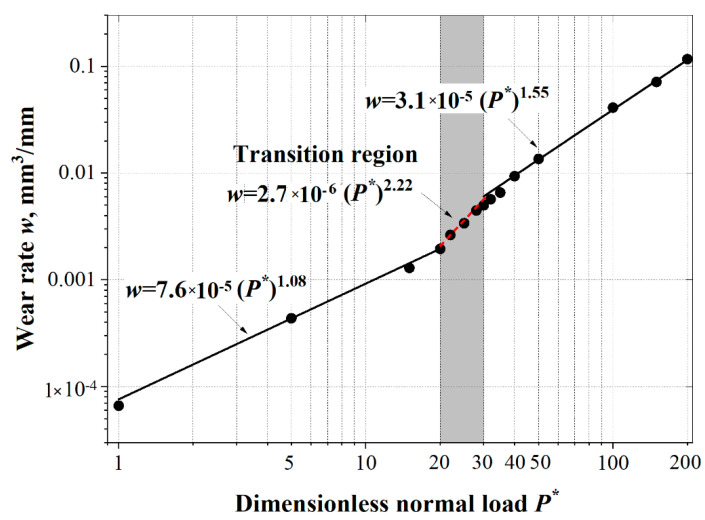
The effect of dimensionless normal load on wear rate. The mild and severe wear regimes and the transition region between them are found. Taken from [49].

Recently, the authors developed an advanced efficient model [49] based on the model in Ref. [46] by adding the submodel technique. This model consists of a global model, showing the potential location of a fracture, and a submodel covering only the region near the potential fracture with refined mesh. Damage initiation and evolution along with the deletion of failed elements are executed only in the submodel. Such a solving procedure drastically reduces the computing time and resolves the overestimation of interference from element deletion, allowing more parametric study with higher accuracy. The effect of normal loading on wear rate was then studied and two main regimes of mild and severe wear (along with a relatively narrow transition region between them) were found (see Figure 2), which show an almost linear and power-law dependency of wear rate on normal loading, respectively. Such behavior agrees with published experimental observations [50,51,52,53].

It can be summarized here that the FE method succeeded in modeling the formation of wear particles based on physical principles without the use of an empirical wear coefficient. Almost all the investigations presented sheet or flake-like wear particles, inconsistent with the assumption of hemispherical ones in the Archard wear law. The models above with FEM mainly apply to ductile materials using criteria related to equivalent plastic strain $\bar{\varepsilon }$ for damage occurrence. It should be noted that in addition to a long computing time, the FE method has other deficiencies such as handling large element distortion, element deletion for fracture representation, and complex contact (and mixture) of small particles during sliding.

### 3.2. Atomic Simulations

**Figure 3:**
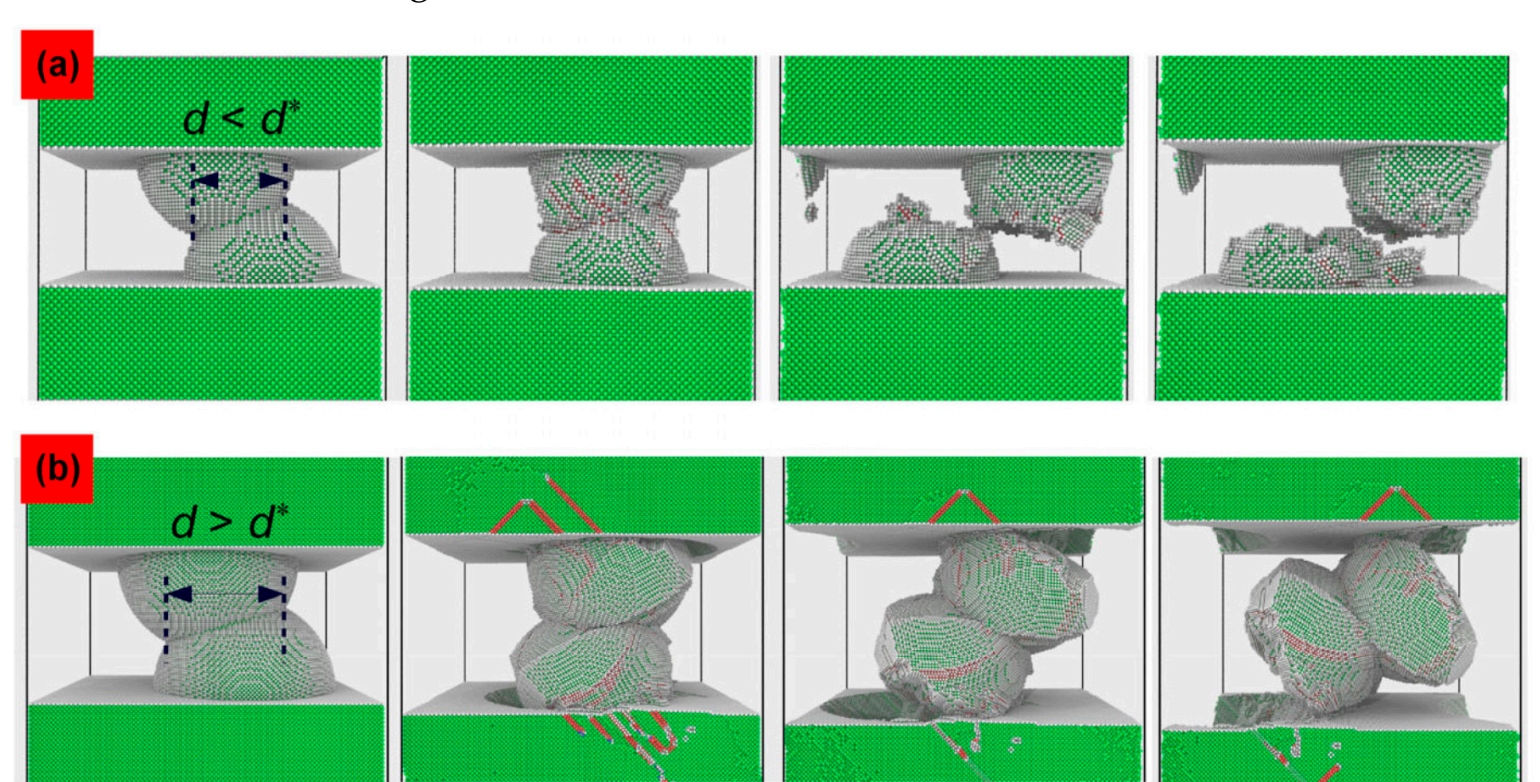
Two adhesive wear mechanisms at the asperity level revealed by atomic simulations, taken from [16]: (**a**) the plastic smoothing mechanism in the absence of a wear debris particle; (**b**) the fracture-induced particle formation mechanism.

Atomic simulations with molecular dynamics attracted great attention in the past decades in nanotribology, which enables insight into the molecular mechanisms of friction and wear [54,55], as that in experiments by atomic-force microscopy (AFM). An important advancement in understanding adhesive wear mechanisms came from the work of Aghababaei et al. [56] in 2016 using coarse-grained atomic simulations. They were inspired by the work of Rabinowicz [4,29] on the minimum size of loose debris particles and they found a characteristic length scale that controls the adhesive wear mechanisms at asperity level. This length scale provides a critical adhesive junction size d* where junction sizes below d* produce plastic flattening of the asperity and above d* produce brittle fracture-induced wear. As shown in Figure 3a below, for a junction size smaller than d*, the strong adhesive forces yield a severe plastic deformation of asperity, and in Figure 3b, for a junction size larger than d*, a debris particle is formed. The expression of d* is given by:

(5) $$d^{*}=f\frac{G}{\tilde{\tau }/2\mu },$$

where *f* is a geometry factor, *G* is the fracture energy, $\tilde{\tau }$ is the shear limit, and *μ* is the shear modulus of the asperity. This work triggered the next extensive studies on the adhesive wear mechanism, using atomic simulations and also other methods.

By systematic atomic simulations, Aghababaei et al. [57] confirmed Archard’s hypothesis that the depth to which the material is worn is proportional to junction size, i.e., that wear rate is proportional to the real contact area at the asperity level (see Section 2.1). They also found that in the presence of strong adhesion, wear particle volume can be better quantified via the work done by the tangential component of the load carried by a junction, instead of the normal force component as used in Archard’s wear law. By further studying the complete process of nucleation, evolution, and detachment of a wear particle during adhesive wear, Aghababaei [58] confirms the hypothesis of wear particle formation by agglomeration of transferred material fragments.

For a case of weak adhesion at the contact interface, Brink et al. [59] found that colliding asperities can either deform plastically to form wear particles or slip along the contact junction surface without significant damage, depending on the material and interface properties and the local slopes of the surfaces. An analytical expression of a refined critical length scale was hence provided, which incorporates the interface properties and roughness parameters. Milanese et al. [60] studied the role of interfacial adhesion on minimum wear particle size and roughness evolution. They found that at short timescales, the surface morphology and not the interfacial adhesion strength dictates the minimum size of wear particles, while at longer timescales, adhesion alters the particle motion and thus the wear rate and the surface morphology.

**Figure 4:**
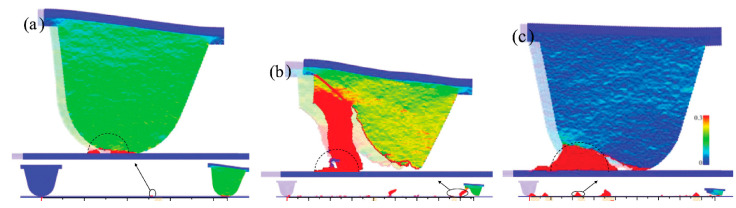
Three observed wear mechanisms: (**a**) atomic cluster detachment; (**b**) cleavage fracture; (**c**) plasticity. The black dashed lines show the size of contact area corresponding to wear volume hypothesized in Archard’s model. Taken from [61].

The macroscopic wear relations described by Archard, i.e., wear volume linearly depends on the normal load and sliding distance, were reviewed by Zhao and Aghababaei et al. [61,62] at the asperity level with systematic long-timescale wear simulations. They found three disparate wear mechanisms (see Figure 4): (1) detachment of atomic clusters (size of detached clusters is smaller than the contact size); (2) abrupt tip fracture (fragment size is bigger than the contact size); (3) plasticity-assisted material removal at the asperity contact (size of detached fragments is comparable to the contact size). The color legend in Figure 4 presents values of the shear component of the deformation gradient tensor. They found that a linear wear relation can be recovered only when the material removal progresses by plastic deformation at the asperity tip, confirming the long-standing theoretical hypothesis made by Archard. This linearity breaks down when cleavage cracking dominates the material removal. Interestingly, this plasticity-assisted detachment seems inconsistent with the finding in Ref. [56], where brittle fracture-induced detachment was shown.

**Figure 5:**
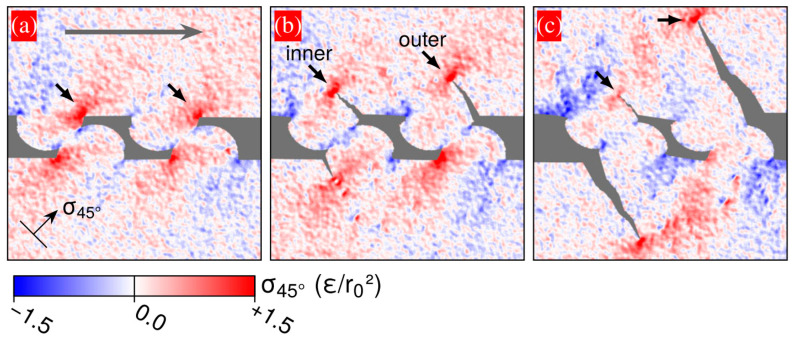
Crack shielding drives joint debris formation: (**a**–**c**) stress component driving the nucleation and propagation of the subsurface cracks, taken from [63].

Based on the study of microcontact on single asperity, Aghababaei et al. [63] also considered the interactions of microcontacts under high normal load, as shown in Figure 5. They found that interaction between subsurface stress fields of neighboring microcontacts promotes a transition from mild to severe wear. In the mild wear regime, asperities are detached in the form of tiny particles, the size of which is comparable to the junction size and, hence, it follows the linear relation of the Archard equation. In the severe wear regime, large wear debris occur due to deep crack propagation below the surface contact. A single particle size in this regime corresponds to the apparent contact area of multiple asperities and, hence, the linear relation of the Archard equation fails.

Yang et al. [64] investigated the wear formation during a single truncated-cone-shaped asperity sliding against a rigid flat using atomic simulation. Two distinct wear mechanisms were observed: low-load atomic wear (isolated debris atoms or clusters) and high-load plastic wear (collective debris formation from plastic flow). This is similar to the findings by Zhao et al. in Refs. [61,62]. A wear mechanism map of atomic/plastic wear was constructed by Yang et al. [64] in the domain of normal stress and adhesion, showing consistency with existing simulation and nanoscale experimental results.

The existing atomic simulations mainly concerned with the formation of wear particles with brittle fracture and plastic deformation for ductile materials are increasingly studied by this method, as was conducted in [61], which used different interatomic potentials to tune the degree of ductility.

### 3.3. Discrete Element Method

**Figure 6:**
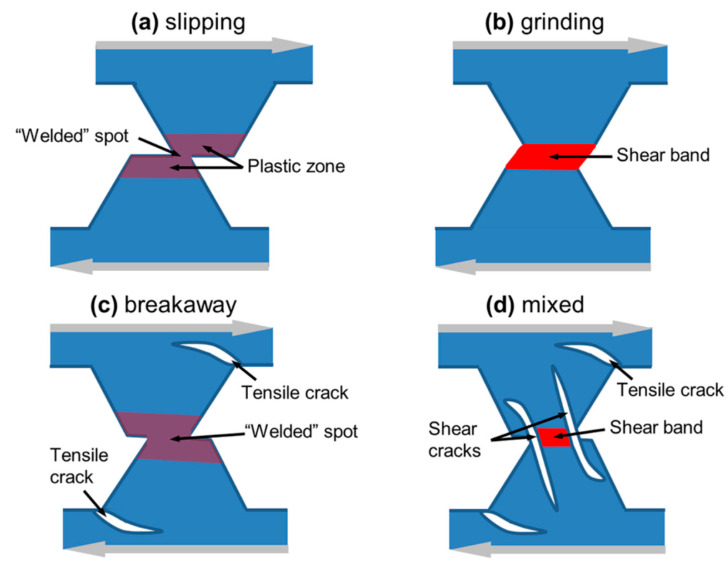
Three wear modes of surface asperities: (**a**) slipping; (**b**) plastic grinding; and (**c**) breakaway; (**d**) mixed modes. Taken from [66].

The particle-based discrete elements method (DEM) is known as a promising and potentially effective tool for the numerical study of complex aspects of macroscale solids’ behavior including multiple fractures, contact interaction and friction, mass transfer, and mixing effects. This provides a powerful method to simulate wear involving formation of transfer fragments and wear particles, as well as the growth of occasional fractures at interfacial elements. Dimaki et al. [65,66] proposed a 2D discrete-element-based model of adhesive wear for elastic-plastic asperity with a trapezoidal shape considering both cohesive and adhesive interactions, as shown in Figure 6. Their model allows for the consideration of both ductile and brittle materials by using the two-parametric failure criterion of Drucker and Prager [67], which includes a parameter *a* governing the brittleness of material. By a detailed study of factors in adhesive wear, they found three main modes of wear: slipping, plastic grinding, and breakaway, as shown in Figure 6a–c, respectively, depending on the material and loading parameters. The occurrence of a particular mode is determined by the combination of two dimensionless material parameters: the ratio of the adhesive stress to the pure shear strength of the material, and the sensitivity parameter of material shear strength to local pressure. The case study map of asperity wear modes in the space of these parameters was also constructed.

Pointing out the high computational cost and narrow window of time and length scales of atomic simulations, Pham-Ba and Molinari [68] recently reproduced the key mechanisms observed in atomic simulations with DEM, using an order of magnitude larger particle diameters and system sizes. The simulations of single asperity wear were successfully reproduced with DEM using a range of particle sizes, validating the coarse-graining procedure.

### 3.4. Phase-Field Approach

Modeling adhesive wear requires accurate prediction of failure and fracture processes, which has proven difficult particularly for full three-dimensional geometries with ductile property. The phase-field approach (PFA), also known as the variational approach to model fracture, is an approach that has recently received much attention in the literature because of its advantages in ease of implementation, robustness, and efficiency [69,70].

Carollo et al. [71] applied the phase field model for fractures to simulate the crack pattern leading to debris formation in a triangular asperity junction model. They found two failure modes: (1) a crack nucleated at the contact boarder (small wear particle), and (2) a crack nucleated at the root of the triangular asperity (large wear particle), depending on the dominant power of the stress-singularity there. Steady-state adhesive wear was observed for triangular asperity with corner of 45°, because in this case the new surface profile created by the fracture presents exactly the same geometry as that of the original one. For the similar triangular asperity junction model, Collet et al. [72] proposed a brittle formulation of the variational phase-field approach to fracture for study of the physical processes of adhesive wear but considered interaction of two neighboring contacts. They found that the failure mechanisms of an adhesive junction can be linked to its geometry. A large debris formation is mostly triggered by tensile stresses, while shear stresses lead to small or no particle formation. With the study for groups of junctions, a classification in terms of macroscopic wear rate was proposed. Brach and Collet [73] extended the work in [72] to elastic-plastic adhesive wear and they observed the transition from perfectly brittle, over quasi-brittle to elastic-plastic wear regimes, as the ductility of the contacting material increases. They proposed a new criterion to discriminate between non-critical and critical asperity contacts, where the former produce negligible wear while the latter lead to significant debris formation. Basically, the phase-field approach, which is able to effectively capture the failure of adhesive junctions, has proven its applicability in modeling wear although based on the approach of continuum mechanics.

## 4. Wear Modeling on Rough Surface

It is possible to model the adhesive wear of a rough surface by integrating the wear at asperity level, which can be obtained, for instance, with one of the methods discussed above. The rough surface can be described statistically or deterministically as was done in contact mechanics of a rough surface [74,75,76]. For wear simulation, with the statistical method it is difficult to accurately describe a surface after wear particles are removed from it, and in particular when removed worn particles have irregular shapes. Continuously tracking the evolving characteristic of an eroding surface and the wear volume (wear rate) becomes more challenging when the wear of long-distance sliding is of interest. However, it should be noted that the statistical method having high efficiency is beneficial in modeling wear of the steady state [77], during which the wear rate (also the statistical parameters of surface topography) can be seen as unchanged. Realizing the disadvantages of the statistical method, the deterministic description, implemented in a numerical model, becomes the primacy consideration for modeling wear of rough surfaces owing to showing details of each contact point. In addition, the deficiencies exist in most numerical methods with deterministic description, such as suffering from a long computing time, which seems the common one in modeling “realistic” rough surfaces, particularly involving wear evolution. The boundary element method (BEM) and semi-analytic method, both based on the analytical fundamental solution, are relatively efficient and hence become the preferred ways to model wear of rough surfaces in the literature.

Frérot et al. [22] applied the criterion of a critical length scale for asperity wear in Ref. [56] into random self-affine (fractal) isotropic surfaces using BEM. The model consists of an elastic half-space in contact with a rigid solid having the fractal rough surface. They presented a physics-based interpretation for the Archard’s wear coefficient *K* by showing its variation in terms of the properties of the interface, surface roughness parameters, and applied load. This work provides an example for possible methods to calculate the wear coefficient of rough surfaces from first principles. Pointing out the unreality of the purely elastic deformation at rough contact interfaces in [22], Frérot et al. [78] further considered the plastic deformation by using both the classical J2 (von Mises) plasticity approach and saturation plasticity model and analyzed the crack nucleation process at the rough contact interface. They found that the saturation plasticity model can only qualitatively reproduce the true contact area and fails to give satisfactory results on local quantities. The J2 model, enhancing the surface tensile stresses, leads to a higher crack nucleation likelihood. This work shows the necessity of an accurate plasticity model for simulation of adhesive wear that includes plasticity. Pham-Ba et al. [79] considered the elastic interactions between nearby asperity-contacts and showed that such interactions can lead to wear particles with volumes that are larger compared to the individuals by enclosing all these nearby asperity-contacts. This work presented an example for possible regime of severe wear in rough surface contact.

According to Equation (5), which was used in modeling wear of rough surfaces in [22,78,79], the value of $d^{*}$ decreases with increasing material hardness (higher shear limit $\tilde{\tau }$), indicating that a harder material can more easily produce wear, in contradiction to experimental observations. Brink et al. [80] realized such a contradiction and developed the parameter-free mechanistic wear model allowing a sliding process based on the work in [22], where only normal contact was calculated. In [80], they found that $d^{*}$ defines not only a minimum wear particle size, but rather a typical wear particle size. Therefore, materials with lower hardness (lower $\tilde{\tau }$) show larger $d^{*}$ and wear more, thereby reconciling the concept of the critical length scale with Archard’s wear law.

**Figure 7:**
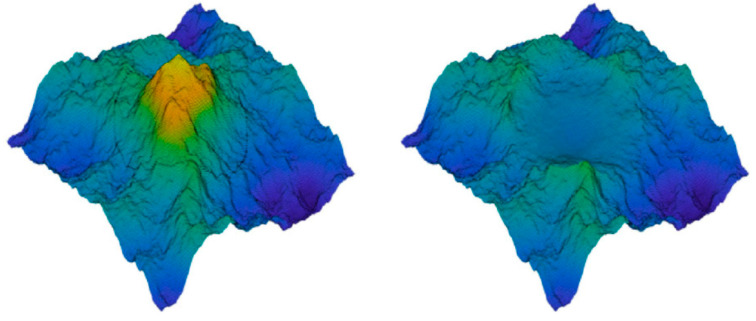
Normal contact between a rough surface and an elastic half-space using BEM: topography of a rough surface before and after detachment and disappearance of a wear particle. Taken from [82].

Popov et al. [81,82] proposed a similar wear model of fractal rough surfaces using BEM, but with a different definition of an asperity, and hence, the criterion at asperity level were not needed by introducing an asperity-free wear criterion (generalization of the Rabinowicz criterion). This was conducted by probing each surface point with circles of varying diameter, and when the Rabinowicz criterion (see Equation (4)) is satisfied, a wear particle forms at this position with this diameter (see Figure 7). With this model, they found two wear regimes: the first is a settling type, without further wear after the topography was modified. The other showed continuously proceeding wear, depending on the contact conditions. They also found a power-law dependency of the wear volume on the normal load for the first wear regime, which is similar to that found in Ref. [49].

Cao et al. [83] proposed a different fracture-induced adhesive wear criterion to predict local wear of material using a semi-analytical method, which also has an advantage in efficiency. This wear criterion is based on the idea that the input frictional work continually leads to nucleation and propagation of subsurface cracks, and hence the energy needed to separate local materials from the substrate decreases. Wear at a surface point occurs only when the work of adhesion exceeds or equals the increase in surface energy if detachment of the material particle occurs. In this criterion, the effects of work of adhesion, surface energy degradation, and also the stochastic distributions of these physical properties of materials on adhesive wear were considered. Then, the wear criterion was coupled with the deterministic mixed lubrication theory to predict the wear process of mixed lubricated point contact, showing the morphology evolution of surfaces.

Shilko et al. [84] presented a 2D discrete-element-based wear model for ductile materials with a cold welding mechanism, allowing the formation of wear particles and a third body during the friction of rough metal surfaces. They found that the behaviors of friction and wear of ductile materials qualitatively differ from those of brittle materials (low surface energy), which more easily produce third body fragments. Based on the numerical results, they emphasized the importance of tangential contact in modeling wear of rough surfaces where a third body plays an important role in friction and wear. This idea was also similarly indicated by Greenwood in Ref. [85].

The key characteristics of the numerical methods reviewed above for modeling the wear of asperity and rough surfaces are summarized in Table 1. It should be noted that scale and efficiency are only roughly compared, and the actual value should depend on the case.

## 5. Conclusions

It is required to predict adhesive wear based on first principles, avoiding empirical parameters, which lack physical understanding of the wear process. Such a vision has existed for decades and recently much progress has been made due to the development in techniques of computer simulation. This paper reviewed the numerical models for adhesive wear along with the two classic theories of the Archard wear law and Rabinowicz’ criterion.

The multiscale nature of the wear problem requires the understanding of the wear mechanism starting from the asperity level. The finite element method, as one of the most long-standing methods in modeling wear, brought new knowledge in the adhesive wear mechanism at the asperity level. Using the realistic elastic-plastic material behavior, the most relevant work with FEM predicted the flake-like wear particle at the asperity tip. The major limitations of the FEM include its computing efficiency in modeling larger scales (in time, and in space, e.g., multi-asperity) of adhesive wear and the difficulties in handling large element distortion, element deletion for fracture representation, and complex contact (and mixture) of small particles during sliding.

Atomic simulations based on molecular dynamics revealed a critical length scale for wear particle formation and this criterion has attracted extensive attention in recent years, leading to a series of works and new findings. The interaction of neighboring asperity contacts was found to be crucial in the wear regime transition from mild to severe. Most of the existing work focused on the brittle fracture-induced wear particle while the wear mechanism due to ductile fracture is not equally understood. The discrete element method and the newly appearing phase-field approach also present promising application in this field.

Modeling adhesive wear of rough surfaces in the literature mainly relied on the boundary element method and semi-analytical method, which provides a contact solution with higher efficiency compared with other methods. However, deficiencies still exist due to the use of simplifications, including the purely elastic material or simplified plasticity model, the Johnson assumption of simplifying the rough–rough contact to flat–rough contact, and the simple disappearance of the detached wear particles without considering their possible following effects in the form of a third-body particle.

Therefore, to address the deficiencies in existing methods, some relevant issues need to be dealt with in future research work of modeling adhesive wear to come closer to the physical mechanism and, hence, accurate prediction. These include the use of more realistic factors, such as the geometry of asperity (dimensions, scales, and profile), material properties with elastic-plastic behavior, and representativeness of rough surfaces [86]. In addition, improving the computing efficiency is of a great importance in almost every numerical method.

The underlying problem in studying wear is the multiplicity of wear mechanisms [85]. Even only for adhesive wear, different criteria were proposed for different cases, and it is expected that more criteria will appear in future studies for adhesive wear and also other wear types, e.g., a recently proposed criterion for abrasive wear [87]. The new criteria could be obtained by theoretical derivation, but more likely by summarizing numerical solutions. This emphasizes the great importance of summarizing and analyzing to avoid those numerical simulations becoming “black boxes” where the mechanisms behind the complex numerical results are lost.

## Figures and Tables

**Table 1:** Summary of wear modeling of asperity and rough surfaces.

	Methods	Scale	Geometry	Material Properties	Basis (Criterion) of Wear	Efficiency	Major Insights
Asperity contact	FEM [32,34,41,44,46,49] etc.	+++	Wedge, Sphere	Elastic, Elastic-plastic	LEFM, maximum shear strain, failure criteria, etc.	+	Morphology of wear particle
	DEM [65,66]	++	Trapezoid			++	
	PFA [71,72,73]	+++	Triangle			+++	
	MD [56,57,58,59,60,61,62]	+	Sphere etc.		Interatomic potentials	+	Critical junction size
Rough surfaces	BEM [22,78,79,80,81,82]	Fractal surface		Elastic (Elastic-plastic [78,84])	Critical junction size	+	New understanding of *K*
	DEM [84]				Fracture mechanics	+++	Formation of third body
	SAM [83]	Gauss surface		Elastic	Energy criterion	++	Surface evolution in long sliding distance

The number of symbol “+” represents the level of how much larger or higher it is.

## Data Availability

Not applicable.
